# Th1 cells downregulate connexin 43 gap junctions in astrocytes via microglial activation

**DOI:** 10.1038/srep38387

**Published:** 2016-12-08

**Authors:** Mitsuru Watanabe, Katsuhisa Masaki, Ryo Yamasaki, Jun Kawanokuchi, Hideyuki Takeuchi, Takuya Matsushita, Akio Suzumura, Jun-ichi Kira

**Affiliations:** 1 Department of Neurology, Neurological Institute, Graduate School of Medical Sciences, Kyushu University, Fukuoka, 812-8582, Japan; 2 Department of Neuroimmunology, Research Institute of Environmental Medicine, Nagoya University, Nagoya, 464-8601, Japan; 3 Institute of Traditional Chinese Medicine, Suzuka University of Medical Science, Suzuka, 510-0226, Japan; 4 Department of Neurology and Stroke Medicine, Yokohama City University Graduate School of Medicine, Yokohama, 236-0004, Japan

## Abstract

We previously reported early and extensive loss of astrocytic connexin 43 (Cx43) in acute demyelinating lesions of multiple sclerosis (MS) patients. Because it is widely accepted that autoimmune T cells initiate MS lesions, we hypothesized that infiltrating T cells affect Cx43 expression in astrocytes, which contributes to MS lesion formation. Primary mixed glial cell cultures were prepared from newborn mouse brains, and microglia were isolated by anti-CD11b antibody-conjugated magnetic beads. Next, we prepared astrocyte-rich cultures and astrocyte/microglia-mixed cultures. Treatment of primary mixed glial cell cultures with interferon (IFN) γ, interleukin (IL)-4, or IL-17 showed that only IFNγ or IL-17 at high concentrations reduced Cx43 protein levels. Upon treatment of astrocyte-rich cultures and astrocyte/microglia-mixed cultures with IFNγ, Cx43 mRNA/protein levels and the function of gap junctions were reduced only in astrocyte/microglia-mixed cultures. IFNγ-treated microglia-conditioned media and IL-1β, which was markedly increased in IFNγ-treated microglia-conditioned media, reduced Cx43 protein levels in astrocyte-rich cultures. Finally, we confirmed that Th1 cell-conditioned medium decreased Cx43 protein levels in mixed glial cell cultures. These findings suggest that Th1 cell-derived IFNγ activates microglia to release IL-1β that reduces Cx43 gap junctions in astrocytes. Thus, Th1-dominant inflammatory states disrupt astrocytic intercellular communication and may exacerbate MS.

Connexins (Cxs) are a family of vertebrate proteins that form gap junction (GJ) channels, the major intercellular channel that facilitates direct signalling between cytoplasmic compartments of adjacent cells. A GJ consists of a pair of hemichannels, each of which is a hexameric cluster of Cxs. Various tissues and cell types exhibit characteristic Cx expression profiles. In the central nervous system (CNS), there are abundant GJs between adjacent astrocytes (A/A junctions) and between oligodendrocytes and astrocytes (O/A junctions)^1^,^2^,^3^. Astrocytes are functionally coupled to adjacent astrocytes and oligodendrocytes by GJs and form the “glial syncytium” that maintains the homeostasis of glial and neuronal cells^4^. Cx43 is regarded as the primary astrocytic GJ protein^5^,^6^. Cx43 is diffusely expressed in the fine processes of cortical astrocytes in grey matter^7^. In white matter, Cx43 expression levels are lower than in grey matter, and Cx43 is present in the foot processes of perivascular astrocytes^7^,^8^,^9^.

Multiple sclerosis (MS) is an inflammatory demyelinating disease of the CNS. The pathological hallmark of MS is demyelinating plaques with relatively preserved axons, suggesting that autoimmune responses preferentially target CNS myelin. We previously reported early and extensive loss of astrocytic Cx43 in active white matter lesions of MS, neuromyelitis optica (NMO), and Baló’s concentric sclerosis (BCS) pateints^10^,^11^,^12^. It has been suggested that early disruption of cell-to-cell communications among glial cells may have a crucial role in the development of demyelinating plaques^12^,^13^,^14^. Perivascular lymphocytic cuffing mainly consisting of T cells has been observed significantly more frequently in active demyelinating lesions with Cx43 loss^11^. Moreover, Cx43 loss is significantly associated with a rapidly progressive disease course, culminating in death^11^. Although some proinflammatory factors have been reported to reduce astrocytic expression of Cx43 *in vitro*^15^, the mechanisms of Cx43 loss remain to be elucidated in demyelinating diseases. Because it is widely accepted that autoimmune T cells are involved in the pathogenesis of MS and experimental autoimmune encephalomyelitis (EAE), an animal model of MS^16^, we hypothesized that infiltrating T cells might alter Cx43 protein levels in astrocytes and contribute to MS lesion extension. In this study, we investigated whether CD4^+^ T cells, such as T helper (Th) 1, Th17, or regulatory T (Treg) cells, directly or indirectly influence Cx43 protein levels in astrocytes using a primary glial cell culture system.

## Results

### Cell types in mixed or purified glial cell cultures

Primary mixed glial cell cultures were prepared from the brains of newborn C57BL/6 J mice. Primary mixed glial cell cultures contained 36.6 ± 11.1% ionized calcium-binding adapter molecule-1 (Iba-1)-positive microglia (five independent experiments), and the remaining cells were almost all glial fibrillary acidic protein (GFAP)-positive astrocytes (Supplementary Fig. S1a). We detected Cx43 on the surface of astrocytes by immunocytochemistry (Supplementary Fig. S1b). Cx30 was not detected in these cultures (Supplementary Fig. S1c). Microglia were isolated from these primary mixed glial cell cultures by anti-CD11b antibody (Ab)-conjugated magnetic beads. Microglial cultures contained >90% Iba-1-positive cells (94.6 ± 2.8%, five independent experiments) (Supplementary Fig. S1d). Next, we prepared astrocyte-rich cultures and astrocyte/microglia-mixed cultures (Supplementary Fig. S1e–f). Astrocyte-rich cultures contained <2% Iba-1-positive cells (0.7 ± 0.7%, nine independent experiments). Astrocyte/microglia-mixed cultures contained 38.2 ± 2.1% (five independent experiments) Iba-1-positive cells when they were used for the following experiments. All of these cultures contained no NeuN-positive cells (neurons), <1% Nogo-A-positive cells (oligodendrocytes), and <1% neuron-glial antigen 2 (NG2)-positive cells (oligodendrocyte precursors) (Supplementary Fig. S1g–i).

### Interferon (IFN) γ downregulates Cx43 protein levels in mixed glial cell cultures

**Figure 1:**
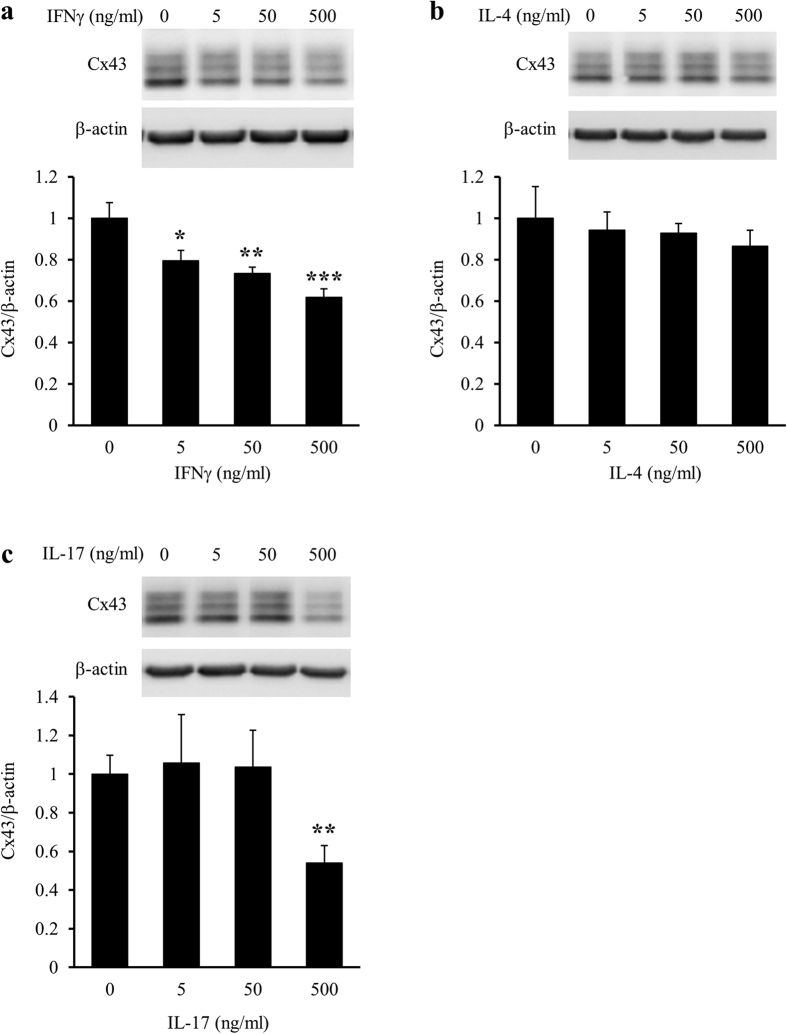
Effects of IFNγ, IL-4, and IL-17 treatments on Cx43 protein levels in primary mixed glial cell cultures. Primary mixed glial cell cultures were treated with the vehicle (0) or 5, 50, and 500 ng/ml IFNγ (**a**), IL-4 (**b**), or IL-17 (c) for 24 h. Cx43 protein levels in primary mixed glial cell cultures were assessed by western blotting. β-actin was used as a loading control. Cx43/β-actin of the vehicle treatment was set as 1. Data are presented as the mean ± s.d. (n = 4, *p < 0.05, **p < 0.01, and ***p < 0.001, compared with the vehicle-treated control by one-way ANOVA followed by Dunnett’s multiple comparison test). Full-length blots are presented in Supplementary Fig. S6.

Primary mixed glial cell cultures were treated with recombinant mouse IFNγ, interleukin (IL)-4, and IL-17, which are produced mainly by Th1, Th2, and Th17 cells, respectively, at concentrations of 0 (control), 5, 50, or 500 ng/ml for 24 h. Western blotting revealed that IFNγ reduced Cx43 protein levels in a dose-dependent manner, and that IL-17 at the highest concentration only (500 ng/ml) reduced Cx43 protein levels, whereas IL-4 did not affect Cx43 protein levels at any tested concentration (Fig. 1).

### IFNγ decreases astrocytic Cx43 protein and mRNA levels only in the presence of microglia

**Figure 2:**
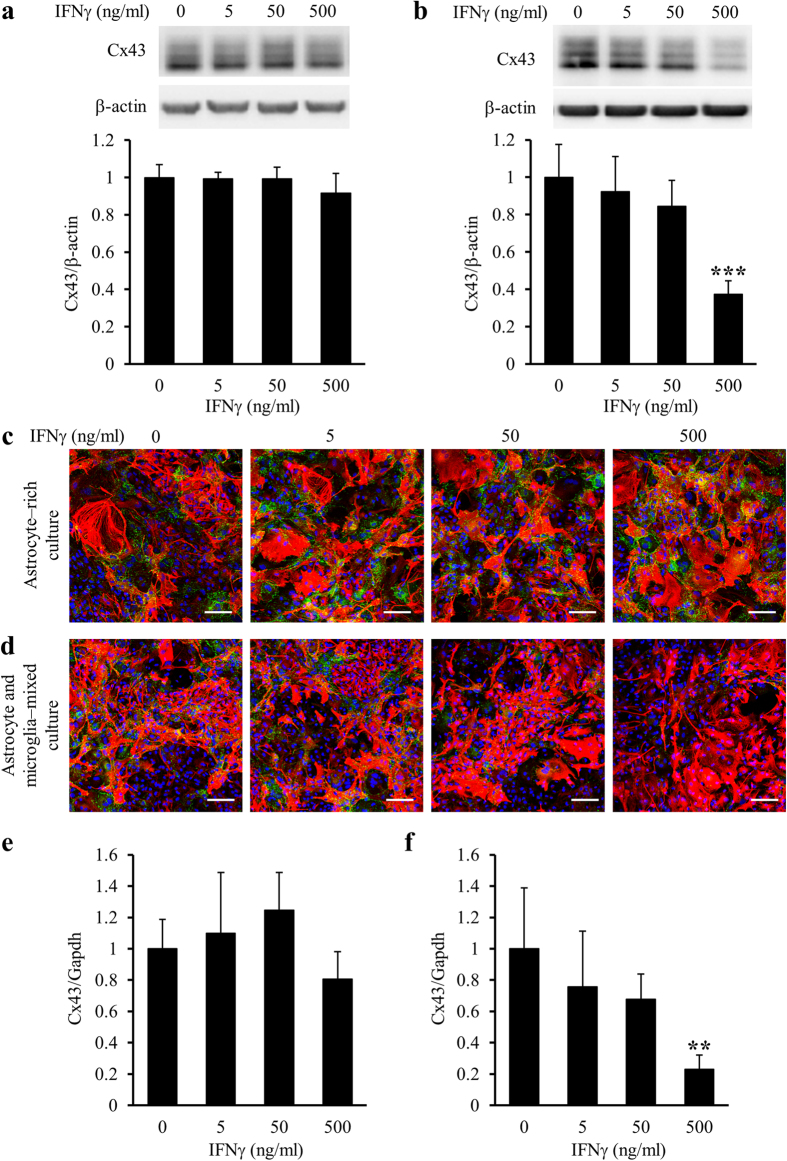
Difference in the effects of IFNγ on Cx43 protein and mRNA levels in the presence or absence of microglia in cultures. Astrocyte-rich cultures (**a,c,e**) and astrocyte/microglia-mixed cultures (**b,d,f**) were treated with the vehicle (0) or 5, 50, and 500 ng/ml IFNγ for 24 h. (**a,b**) Cx43 protein levels were assessed by western blotting. β-actin was used as a loading control. Cx43/β-actin of the vehicle treatment was set as 1. Data are presented as the mean ± s.d. (n = 4, ***p < 0.001, compared with the vehicle-treated control by one-way ANOVA followed by Dunnett’s multiple comparison test). Full-length blots are presented in Supplementary Fig. S7. (**c,d**) Cx43 protein levels were assessed by immunocytochemical staining. Fixed cells were immunostained for Cx43 (green) and GFAP (red), and counterstained with DAPI (blue). Scale bars: 100 μm. (**e,f**) Cx43 (*Gja1*) mRNA levels were analysed by quantitative real-time RT-PCR. The expression of Cx43 mRNA was normalized to that of Gapdh mRNA as an internal control. Cx43/Gapdh of the vehicle treatment was set as 1. Data are presented as the mean ± s.d. (n = 4, **p < 0.01, compared with the vehicle-treated control by one-way ANOVA followed by Dunnett’s multiple comparison test).

**Figure 3:**
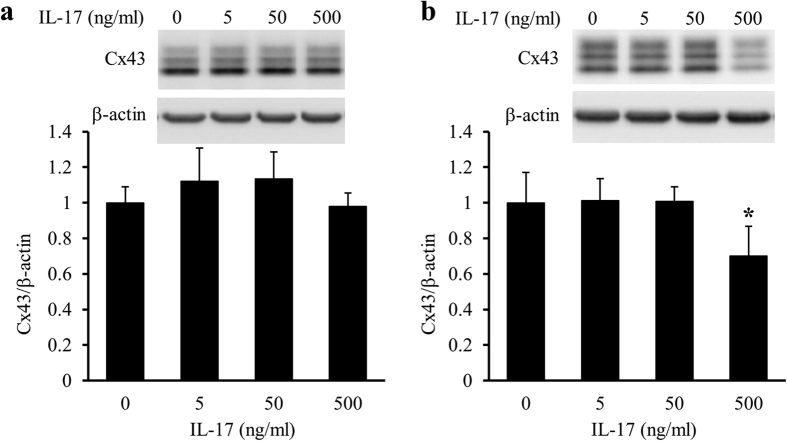
Difference in the effects of IL-17 on Cx43 protein levels in the presence or absence of microglia in cultures. Astrocyte-rich cultures (**a)** and astrocyte/microglia-mixed cultures (**b**) were treated with the vehicle (0) or 5, 50, and 500 ng/ml IL-17 for 24 h. Cx43 protein levels were assessed by western blotting. β-actin was used as a loading control. Cx43/β-actin of the vehicle treatment was set as 1. Data are presented as the mean ± s.d. (n = 4, *p < 0.05, compared with the vehicle-treated control by one-way ANOVA followed by Dunnett’s multiple comparison test). Full-length blots are presented in Supplementary Fig. S7.

Because primary mixed glial cell cultures contained mainly astrocytes and microglia, we next examined whether IFNγ reduced Cx43 protein and mRNA levels in astrocytes directly or via microglia. Upon treatment of astrocyte-rich cultures with IFNγ for 24 h, the protein and mRNA levels of Cx43 were unchanged (Fig. 2a,c,e). In contrast, Upon treatment of astrocyte/microglia-mixed cultures with IFNγ for 24 h, the reduction of Cx43 protein and mRNA levels was dose dependent (Fig. 2b,d,f). Upon treatment of astrocyte-rich cultures and astrocyte/microglia-mixed cultures with IL-17 for 24 h, only the highest concentration of IL-17 reduced the protein levels of Cx43 in astrocyte/microglia-mixed cultures, but not in astrocyte-rich cultures (Fig. 3). These observations implied that both IFNγ and IL-17 reduced Cx43 protein and mRNA levels in astrocytes via microglia. Because IL-17 only affected Cx43 protein levels at extremely high concentrations, we focused on the effects of IFNγ on glial cells in the following experiments.

### Microglia activated by IFNγ suppress the function of GJs in astrocytes

**Figure 4:**
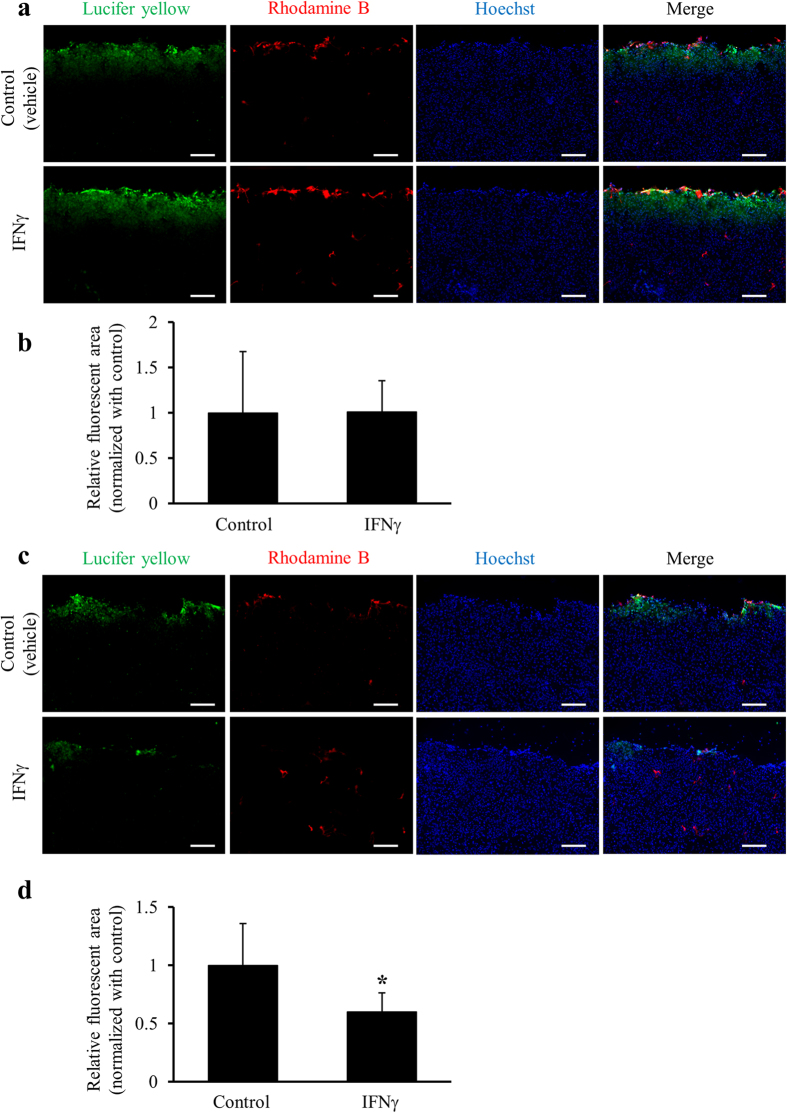
Suppression of the function of GJs by treatment with IFNγ in the presence or absence of microglia. Confluent astrocyte-rich cultures (**a,b**) and confluent astrocyte/microglia-mixed cultures (**c,d**) were treated with the vehicle (control) or 500 ng/ml IFNγ for 24 h. The functional states of GJs were measured by an SLDT assay as described in the Methods. (**a,c**) Representative micrographs show the distribution of Lucifer yellow (green) and Rhodamine B (red) in SLDT assays counterstained with Hoechst 33342 (blue). Scale bars: 500 μm. (**b,d**) Graphs show quantitative data of the dye-spreading areas (rhodamine B-positive areas were subtracted from Lucifer yellow-positive areas) expressed as relative to those in controls (vehicle treatment). Data are presented as the mean ± s.d. (two to three spots per well, n = 4 wells per treatment). *p < 0.05, one-way ANOVA followed by Dunnett’s multiple comparison test.

We next assessed the functional states of GJs using a scrape loading/dye transfer (SLDT) assay. Upon treatment of astrocyte-rich cultures and astrocyte/microglia-mixed cultures with IFNγ for 24 h, the functions of GJs were significantly suppressed in astrocyte/microglia-mixed cultures, but not in astrocyte-rich cultures (Fig. 4).

### Humoural factors secreted from IFNγ-activated microglia decrease Cx43 protein levels in astrocytes

**Figure 5:**
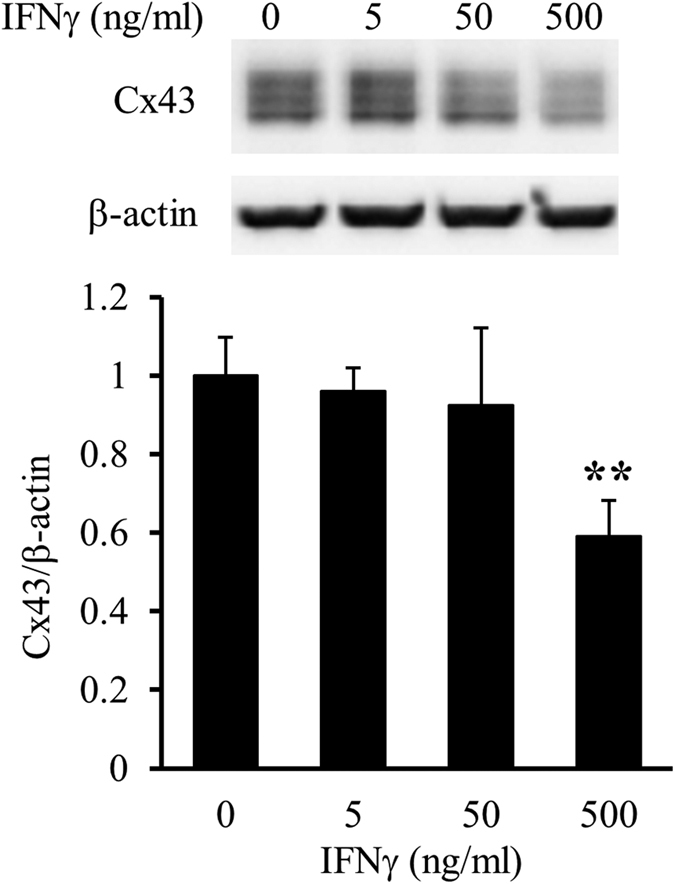
Downregulation of Cx43 protein levels by IFNγ-treated microglia-conditioned media in astrocyte-rich cultures. Isolated microglia were treated with the vehicle (0) or 5, 50, and 500 ng/ml IFNγ for 24 h. IFNγ-treated microglia-conditioned media were collected and used to treat astrocyte-rich cultures for 24 h. Cx43 protein levels were assessed by western blotting. β-actin was used as the loading control. Cx43/β-actin of control (vehicle-treated microglia conditioned medium) was set as 1. Data are presented as the mean ± s.d. (n = 4, **p < 0.01, compared with the vehicle-treated control by one-way ANOVA followed by Dunnett’s multiple comparison test). Full-length blots are presented in Supplementary Fig. S8.

Next, microglial cultures were treated with IFNγ and their supernatants were collected after 24 h (IFNγ-treated microglia-conditioned media). Resting microglia had a rod shape, whereas IFNγ-treated microglia showed morphological changes including a large and round, amoeboid shape (Supplementary Fig. S2). When IFNγ-treated microglia-conditioned media were applied to astrocyte-rich cultures, astrocytic Cx43 protein levels were significantly downregulated after 24 h (Fig. 5). These findings suggest that IFNγ activates microglia, and that humoural factors secreted from activated microglia decrease Cx43 protein levels in astrocytes.

### Identification of humoural factors that decrease Cx43 protein levels in astrocytes

**Figure 6:**
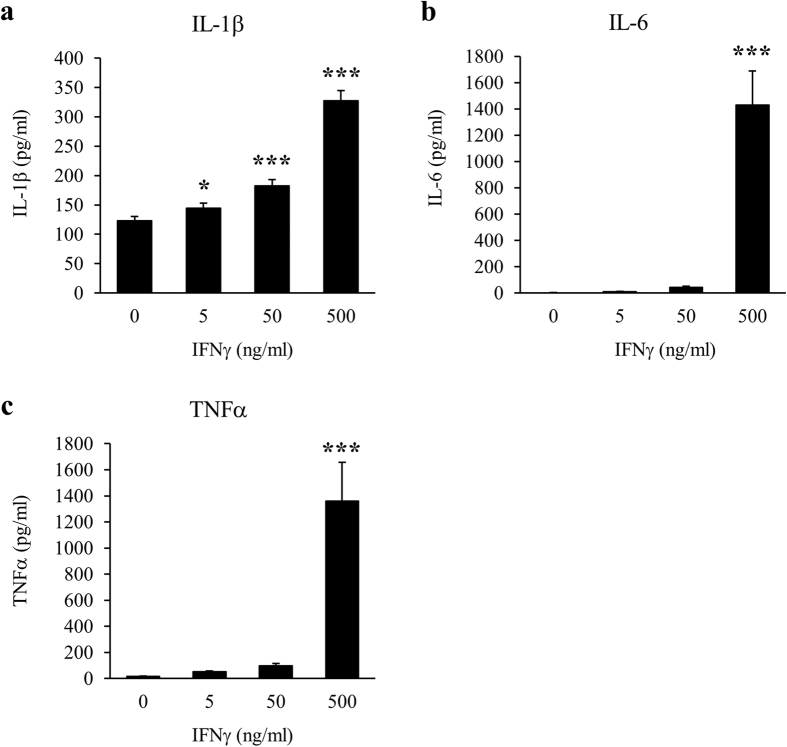
Proinflammatory cytokine concentrations in IFNγ-treated microglia-conditioned media. Isolated microglia were treated with the vehicle (0) or 5, 50, and 500 ng/ml IFNγ for 24 h. IFNγ-treated microglia-conditioned media were collected and several cytokine concentrations were measured using the Bio-Plex Multiplex System. The expression levels of (**a**) IL-1β, (**b**) IL-6, and (**c**) TNFα are shown in each graph. Data are presented as the mean ± s.d. (n = 4, *p < 0.05, **p < 0.01, and ***p < 0.001, compared with the vehicle-treated control by one-way ANOVA followed by Dunnett’s multiple comparison test).

**Figure 7:**
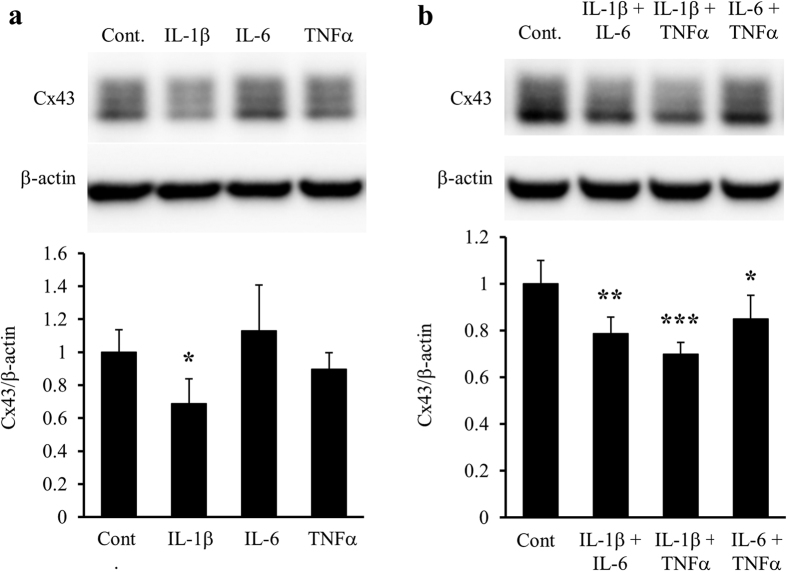
Effects of IL-1β, IL-6, and TNFα on Cx43 protein levels in astrocyte-rich cultures. Astrocyte-rich cultures were treated with 300 pg/ml IL-1β, 1400 pg/ml IL-6, or 1300 pg/ml TNFα alone (**a**) or in combinations (**b**) for 24 h. Cx43 protein levels were evaluated by western blotting. β-actin was used as a loading control. Cx43/β-actin of vehicle treatment (control, shown as “Cont.”) was set as 1. Data are presented as the mean ± s.d. (n = 5, *p < 0.05, **p < 0.01, and ***p < 0.001, compared with the control by one-way ANOVA followed by Dunnett’s multiple comparison test). Full-length blots are presented in Supplementary Fig. S8.

To identify the humoural factors secreted from activated microglia, we focused on several cytokines and chemokines, and measured their concentrations in IFNγ-treated microglia-conditioned media using a Bio-Plex Multiplex System. IFNγ treatment significantly enhanced microglial secretion of all measured cytokines and chemokines in a dose-dependent manner (Fig. 6 and Supplementary Fig. S3). In particular, we focused on IL-1β, IL-6, and tumour necrosis factor (TNF) α that were present in IFNγ-treated microglia-conditioned media at high concentrations (peak values: >100 pg/ml) and have been reported as representative proinflammatory cytokines secreted from activated microglia^17^,^18^,^19^. We treated astrocyte-rich cultures with either IL-1β, IL-6, or TNFα for 24 h. IL-1β significantly reduced Cx43 protein levels in astrocytes in a dose-dependent manner (Fig. 7a, Supplementary Fig. S4). However, upon treatment of astrocyte-rich cultures with combinations of these cytokines, not only IL-1β and IL-6 or TNFα, but also IL-6 and TNFα reduced astrocytic Cx43 protein levels, although IL-6 or TNFα alone did not change Cx43 protein levels (Fig. 7b). Therefore, the main humoural factor secreted from microglia, which decreases Cx43 protein levels in astrocytes, is IL-1β, whereas only IL-6 and TNFα in combination decreases astrocytic Cx43 protein levels.

### Culture supernatants of Th1 cells downregulate Cx43 protein levels in mixed glial cell cultures

**Figure 8:**
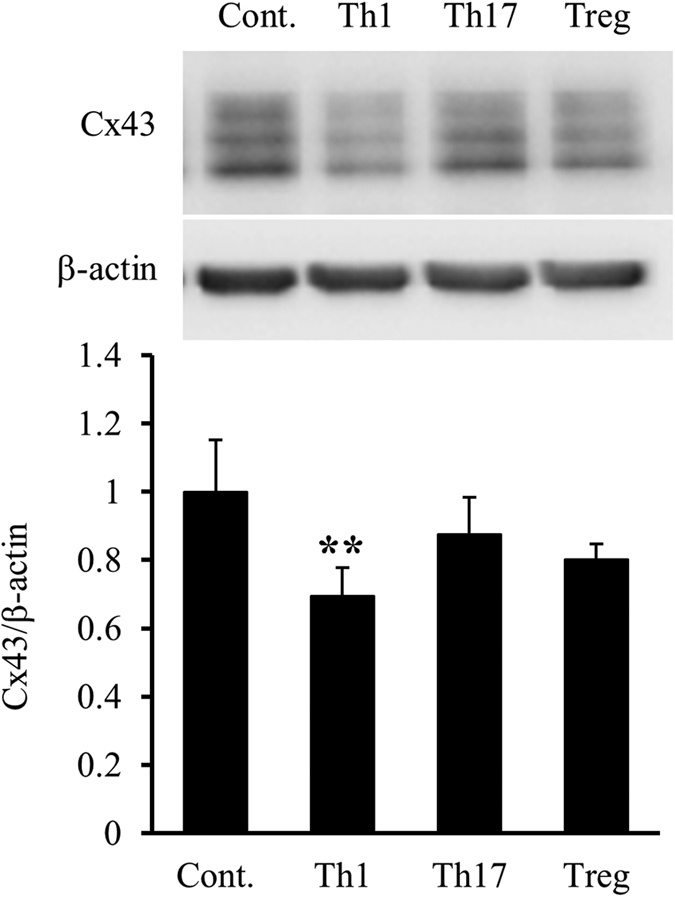
Reduction of Cx43 protein levels in astrocytes induced by the Th1 cell culture supernatant. Mixed glial cell cultures were treated with conditioned media of Th1, Th17, and Treg cells for 24 h. Cx43 protein levels were evaluated by western blotting. β-actin was used as a loading control. Cx43/β-actin of mixed glial cells without treatment (control, shown as “Cont.”) was set as 1. Data are presented as the mean ± s.d. (n = 4, **p < 0.01, compared with the control by one-way ANOVA followed by Dunnett’s multiple comparison test). Full-length blots are presented in Supplementary Fig. S9.

Next, we examined whether Th1 cells reduced Cx43 protein levels in mixed glial cell cultures. Stocks of conditioned media from Th1, Th17, and Treg cells differentiated from naïve T cells *in vitro* contained 444.6, 0.1, and 3.2 ng/ml IFNγ, respectively, as measured by enzyme-linked immunosorbent assay (ELISA). IFNγ was not detected in glial medium (GM) or complete RPMI medium. Primary mixed glial cell cultures were treated with conditioned media from individual T cell subsets for 24 h, and then changes in Cx43 protein levels were quantified by western blotting. As shown in Fig. 8, only Th1 cell-conditioned medium significantly reduced Cx43 protein levels in astrocytes (*p* = 0.0038). These findings suggest that IFNγ derived from Th1 cells activates microglia to release IL-1β, the main factor in the reduction of Cx43 protein levels in astrocytes.

## Discussion

In this study, we demonstrated that IFNγ activated microglia to release IL-1β that reduced astrocytic Cx43 mRNA and protein levels, and functionally inhibited GJs in astrocytes. Although IL-1β secreted from microglia activated by IFNγ appeared to be the main factor in the reduction of Cx43 in astrocytes, other proinflammatory cytokines in concert, such as TNFα and IL-6, also reduced Cx43. Significant downregulation of astrocytic Cx43 was also induced by humoural factors secreted from Th1 cells, particularly IFNγ. Unexpectedly, high concentrations of IL-17 also diminished Cx43 protein levels in astrocytes, but only in the presence of microglia.

It was previously reported that functionally coupled astroglial cells determined by a dye injection method are decreased in astrocyte/microglia cocultures from newborn rat brains in the presence of 5% microglia following administration of either TNFα, IL-1β, or IFNγ^20^. However, it was unclear which cytokines were mainly responsible for Cx43 down-modulation and whether these cytokines acted directly or indirectly on astrocytes. In the present study, we clearly demonstrated that IFNγ indirectly decreased astrocytic Cx43 protein levels through activation of microglia and the subsequent release of IL-1β. Our findings are consistent with a previous study showing that humoural factors secreted from microglia activated by lipopolysaccharide (LPS) stimulation reduce Cx43 expression in astrocytes^18^. Although IL-1β and TNFα secreted from LPS-stimulated microglia have been reported to be the main factors that inhibit Cx43 expression and functions of GJs in astrocytes^18^, in our study, IL-1β alone decreased Cx43 protein levels and TNFα had no additional effect on the Cx43 protein reduction induced by IL-1β treatment. These data are in accordance with previous studies that reported IL-1β downregulates the expression of Cx43 in astrocyte-rich cultures^21^,^22^. However, our present data differ from previous reports regarding the effects of proinflammatory cytokines on glial cells^23^,^24^. Haghikia *et al*. reported that TNFα inhibits the function of GJs in astrocyte/microglia co-cultures containing 5% microglia derived from rat brain^23^. Zhang *et al*. reported that IFNγ and TNFα, but not IL-1β, directly reduce Cx43 expression and suppress the function of GJs in newborn rat-derived spinal astrocyte cultures, in which microglia were removed by shaking off, and >95% of the remaining cells were positive for GFAP^24^. In accordance with a previous report^25^, we also found that the shaking-off method alone was insufficient to fully remove microglial cells, as determined by Iba-1 staining. Therefore, we believe that microglia should be isolated from mixed glial cell cultures using anti-CD11b antibody-conjugated magnetic beads to purify astrocytes maximally^26^,^27^, and that differences in microglial removal methods may be partly responsible for the discrepancy among studies. Differences in animal species, cell sources, and culture conditions might also be causes for the inconsistency, because astrocytes demonstrate inter-species and regional morphological, molecular, and physiological heterogeneity^28^,^29^.

Our study indicates that decreased Cx43 protein levels in astrocytes were in part attributable to a reduction of Cx43 mRNA transcription caused by IFNγ-activated microglia. The detailed intracellular signalling pathway that regulates Cx43 expression remains to be elucidated in astrocytes. Recently, intracellular signalling pathways, including c-Jun N-terminal kinase (JNK), nuclear factor-κB (NF-κB), and phosphatidylinositol 3-kinase, were reported to regulate the expression of Cx43 in astrocytes^24^,^30^,^31^. In addition, the JNK-dependent ubiquitin-proteasome system was reported to be involved in regulating the protein levels of Cx43 through degradation^31^,^32^. Because IL-1β activates JNK and NF-κB pathways in astrocytes^33^, the decrease in Cx43 protein levels might be partly caused by a reduction in Cx43 mRNA transcription through JNK and NF-κB pathways and increased degradation by activation of the JNK pathway via IL-1β secreted from IFNγ-activated microglia.

We and others have reported significant increases in IFNγ-producing T cells in the cerebrospinal fluid (CSF) of MS patients, and the presence of IFNγ-positive lymphocytes in MS lesions^34^,^35^,^36^. Notably, IFNγ injection causes MS relapses^37^. IFNγ production also correlates with exacerbation of neurological symptoms^38^. These findings indicate active involvement of IFNγ-producing Th1 cells in MS. However, the mechanisms of how these Th1 cells contribute to demyelinating lesion formation are not completely resolved. How huge demyelinating lesions occasionally develop despite the presence of perivascular inflammatory cell infiltration occurring in limited areas around vessels is also unclear. We previously reported extensive loss of Cx43 in MS and NMO lesions, and that cases with extensive Cx43 loss more frequently have a malignant course culminating in death within 2 years after disease onset^11^. It is notable that astrocytic Cx43 expression is lost at the leading edges of concentric lesions in BCS patients, where myelin and myelin proteins are preserved, including oligodendrocytic Cx32 and Cx47^10^. In EAE induced by myelin oligodendrocyte glycoprotein or myelin basic protein, diffuse loss of glial Cxs, such as Cx32, Cx43, and Cx47, occurs in acute lesions^39^,^40^. Based on these observations, we propose that early loss of astrocytic Cx43 may lead to the formation of extensive demyelinating lesions. This notion is supported by the fact that astrocytic Cx43/Cx30 double-knockout mice show widespread white matter pathologies including vacuolated oligodendrocytes, intramyelinic oedema, loss of mature oligodendrocytes, and increased numbers of apoptotic cells^41^.

Astrocytes form glial syncytia by coupling to adjacent astrocytes and oligodendrocytes via Cx43 GJs. This process maintains homeostasis of the CNS^4^. Therefore, early loss of astrocytic Cx43 may promote oligodendrocyte apoptosis by disrupting glial syncytia, resulting in secondary demyelination. Furthermore, the absence of astroglial Cx43/Cx30 weakens blood-brain barrier integrity^42^. Intriguingly, Boulay *et al*. recently reported that astroglial Cx43 controls immune cell recruitment^43^. Thus, downregulation of astrocytic Cx43 may promote infiltration of immune cells into brain parenchyma and propagate inflammatory reactions, especially in the presence of proinflammatory cytokines and chemokines produced by activated microglia, as shown by the current and previous studies^44^,^45^. Taken together, IFNγ-dominant inflammatory states might disrupt astrocytic intercellular communication, which can lead to exacerbation of inflammation and extensive demyelination.

In terms of IL-17, *IL17* mRNA expression, the frequency of Th17 cells, and IL-17 levels increase in the blood and CSF of MS patients^46^,^47^,^48^. Th17 cells are enriched in MS lesions^49^, and both Th17 cells and IL-17 have a pivotal role in the pathogenesis of EAE^50^,^51^. It is interesting that high IL-17 concentrations downregulated the Cx43 protein levels of astrocytes in coculture with microglia in our study. Thus, Th17 cells may also contribute to Cx43 loss in the brain parenchyma where astrocytes and microglia may be exposed to high concentrations of IL-17 when in the close contact with infiltrated Th17 cells.

It was recently reported that two-thirds of CNS-infiltrating Th17 cells express IFNγ in EAE^52^,^53^, and that T cells secreting IL-17 alone or IL-17 and IFNγ infiltrate the CNS prior to the onset of clinical symptoms of EAE, where they may mediate CNS inflammation through microglial activation^54^. It was also reported that IL-17^+^ IFNγ^+^ CD4^+^ T cells are abundant in MS lesions^55^. These IL-17 and IFNγ double-positive cells are regarded as pathogenic Th17 cells that develop under a Th1-prone cytokine milieu and become ex-Th17 cells producing IFNγ but not IL-17^56^,^57^. Therefore, Th17 cells may also contribute to MS lesion formation via IFNγ that effectively reduces Cx43 expression in astrocytes.

In conclusion, we propose that Th1 cell-derived humoural factors, mainly IFNγ, induce microglial activation and the release of IL-1β that downregulates astrocytic Cx43, which might exacerbate the inflammatory processes in demyelinating disorders. Thus, IFNγ and Th1-prone conditions are important targets to prevent development of extensive demyelinating lesions.

## Methods

### Animals

All cultures were prepared using cells from C57BL/6 J mice (Charles River Laboratories Japan, Inc., Yokohama, Japan). The protocols for animal experiments were reviewed and approved by the Committee of Ethics on Animal Experiments at Kyushu University Faculty of Medicine (A25–196, A27–205). All animal experiments were performed in accordance with the Regulations for Animal Experiments defined by the Institutional Animal Care and Use Committee at Kyushu University.

### Glial cell cultures

Primary mixed glial cell cultures were prepared from the brains of newborn C57BL/6 J mice according to a previously described method^58^. Briefly, brains were removed under sterile conditions, and the meninges were carefully removed. The tissue was dissociated by passing through a nylon mesh in Hanks’ balanced salt solution (HBSS; Sigma-Aldrich, Saint Louis, MO, USA) containing 50 U/ml penicillin and 50 μg/ml streptomycin (Gibco, Thermo Fisher Scientific, Waltham, MA, USA) to prevent contamination. After washing with HBSS, the cell suspension was plated in 75 cm^2^ culture flasks at a density of one to two brains per flask in 10 ml of GM. GM consisted of Dulbecco’s Modified Eagle’s Medium (Sigma-Aldrich) supplemented with 10% fetal bovine serum (FBS) (Equitech-Bio, Kerrville, TX, USA), 5 μg/ml bovine insulin (Sigma-Aldrich), and 0.2% glucose. The cells were maintained at 37 °C in a humidified atmosphere containing 5% CO_2_ with three medium changes in the first week and no medium change in the second week to induce the proliferation of microglia. At confluency (12–15 days), mixed glial cells were detached by Accutase (Innovative Cell Technologies, San Diego, CA, USA) treatment and replated. After 5–10 days of culture, mixed glial cell cultures that had reached 100% confluence were used for experiments.

We also generated astrocyte-rich cultures and microglial cultures from primary mixed glial cell cultures using magnetic-activated cell sorting (MACS, Miltenyi Biotec, Bergisch Gladbach, Germany). Mixed glial cell cultures were prepared as described above, and glial cells were detached by Accutase treatment at day 12–15. The cell suspension was washed and resuspended in 10 ml MACS Separation Buffer (Miltenyi Biotec). After filtration through a 70 μm pore filter, the cell suspension was centrifuged at 300 × *g* for 5 min at 4 °C. Then, the cells were separated into CD11b-positive and -negative fractions using CD11b MicroBeads (microbeads conjugated to a monoclonal rat anti-mouse CD11b Ab; Miltenyi Biotec) according to the manufacturer’s protocol with some modification^26^,^27^. Briefly, 1 × 10^7^ cells were resuspended in 90 μl of separation buffer and 10 μl of CD11b MicroBeads, and incubated at 4 °C for 30 min with gentle mixing every 10 min. Then, the cells were washed and resuspended in 500 μl of separation buffer per 1 × 10^8^ cells. The cell suspension was applied to an LS column (Miltenyi Biotec) fitted into the QuadroMACS™ cell separator (Miltenyi Biotec), and then the cells were separated into CD11b-negative and -positive fractions. The astrocyte-enriched fraction (astrocyte-rich culture), corresponding to the CD11b-negative fraction, was resuspended in GM before plating. After 7–10 days, astrocyte-rich cultures that reached 100% confluence were used for experiments. The CD11b-positive fraction containing microglia was plated in GM and used for experiments after 24 h (microglial culture). CD11b-negative and -positive fractions were mixed at a ratio of 3:1 (astrocyte/microglia-mixed culture). After 7–10 days, mixed cultures that reached 100% confluence were used for experiments.

### Helper T cell differentiation

Spleens were aseptically removed from C57BL/6 J mice (8–10 weeks old), and splenocytes were dissociated into single cells. After red blood cell lysis, naïve CD4^+^ T cells were isolated from the splenocytes using a Naïve CD4^+^ T Cell Isolation Kit (Miltenyi Biotec) according to the manufacturer’s protocol and suspended in complete RPMI medium [RPMI-1640 medium supplemented with 2 mM L-glutamine (Sigma-Aldrich), 1 mM sodium pyruvate (Gibco, Thermo Fisher Scientific), 50 μM 2-mercaptoethanol, 50 U/ml penicillin, 50 μg/ml streptomycin, and 10% FBS (Cell Culture Bioscience, Lenexa, KS, USA)]. Purified naïve CD4^+^ T cells (1 × 10^5^ cells/well) were seeded in 96-well plates precoated with 5 μg/ml anti-CD3e Ab. The cells were cultured under Th1 (20 ng/ml IL-2, 20 ng/ml IL-12, and 10 μg/ml anti-IL-4 Ab), Th17 [5 ng/ml transforming growth factor (TGF) β, 30 ng/ml IL-6, and 10 μg/ml anti-IFNγ Ab), or Treg (5 ng/ml TGFβ and 20 ng/ml IL-2)-skewing conditions in 200 μl of complete RPMI medium with 2 μg/ml anti-CD28 Ab for 3 days. On day 3, differentiated T cells were collected and washed with complete RPMI medium. Then, the cells were resuspended in complete RPMI medium at 5 × 10^5^ cells/ml and reseeded with 2 μg/ml anti-CD28 Ab in 96-well plates precoated with anti-CD3e Ab for 24 h. On day 4, the T cell culture supernatants were collected and stored at −80 °C before being used for treatment of glial cells. Recombinant mouse IL-2, IL-12, and TGFβ were purchased from R&D Systems (Minneapolis, MN, USA). Recombinant mouse IL-6 was purchased from BioLegend (San Diego, CA, USA). No azide/low endotoxin-grade anti-IL-4 (clone 11B11, rat IgG_1_), anti-IFNγ (clone XMG1.2, rat IgG_1_), anti-CD3e (clone 145-2C11, hamster IgG_1_), and anti-CD28 (clone 37.51, hamster IgG_2_) Abs were purchased from BD Biosciences (Franklin Lakes, NJ, USA).

The purity and differentiation state of naïve CD4^+^ T cells and differentiated T cells were confirmed by flow cytometry. Before staining, Th1 and Th17 cells were stimulated with 25 ng/ml phorbol 12-myristate 13-acetate (Sigma-Aldrich) and 1 μg/ml ionomycin (Sigma-Aldrich) for 5 h, and 10 μg/ml brefeldin A (Sigma-Aldrich) was added for the last 4 h. T cells were washed and blocked with an anti-CD16/32 Ab. Naïve CD4^+^ T cells were stained with anti-CD4 (clone RM4-5), anti-I-A/I-E, anti-CD44, and anti-CD62L Abs. Th1 and Th17 cells were stained with anti-CD4 (clone RM4-5) and anti-I-A/I-E Abs. Treg cells were stained with anti-CD4 (clone GK1.5), anti-I-A/I-E, and anti-CD25 Abs. Differentiated T cells were fixed and permeabilized with Fixation buffer and Permeabilization Wash Buffer (Sony Biotechnology, Champaign, IL, USA) for Th1 and Th17 cells or FOXP3 Fix/Perm buffer Set (Sony Biotechnology) for Treg cells, and then stained intracellularly (anti-IFNγ, anti-IL-4, and anti-IL-17A Abs for Th1 and Th17 cells; anti-mouse/rat Foxp3 Ab for Treg cells). The cells were subsequently analysed by flow cytometry using a Cell Sorter SH-800 (Sony, Tokyo, Japan). Detailed information of the Abs used in flow cytometry is listed in Supplementary Table S1.

The purity of naïve CD4^+^ T cells (CD44^lo^CD62^+^ cells) was typically 93% or higher. Differentiated Th1 and Th17 cells predominantly expressed IFNγ and IL-17, respectively, but rarely expressed IL-4. Most Treg cells expressed both CD25 and Foxp3 (Supplementary Fig. S5).

### Treatment of glial cells

Mixed glial cell cultures, astrocyte-rich cultures, and astrocyte/microglia-mixed cultures were treated with recombinant mouse IFNγ, IL-4, and IL-17 (R&D Systems) diluted in GM for 24 h. Astrocyte-rich cultures were treated with recombinant mouse IL-1β, IL-6 (BioLegend), and TNFα (R&D Systems) diluted in GM for 24 h. The concentrations of these cytokines were 300 pg/ml for IL-1β, 1400 pg/ml for IL-6, and 1300 pg/ml for TNFα based on their concentrations in IFNγ-treated microglia-conditioned medium. Astrocyte-rich cultures were also treated with 0, 180, 240 or 300 pg/ml recombinant mouse IL-1β for 24 h. The concentrations of IL-1β at 180 and 300 pg/ml were approximately equal to those of IL-1β in microglia-conditioned media when microglia were treated with 50 or 500 ng/ml IFNγ for 24 h, respectively. Upon treatment of mixed glial cells with conditioned media from T cells, they were diluted in GM at a ratio of 1:1, and treatments were performed for 24 h.

### Western blotting of Cx43

After all treatments of glial cells, the cells were solubilised in a radioimmunoprecipitation assay buffer containing a protease inhibitor cocktail, 0.5% sodium dodecyl sulfate (Nacalai Tesque, Kyoto, Japan), and PhosSTOP phosphatase inhibitor cocktail (Roche Diagnostics, Mannheim, Germany). The lysates were placed on ice for 30 min and then centrifuged at 4 °C for 10 min at 10,000 × *g*. Supernatants were collected, analysed for protein concentrations using a BCA protein assay kit (Pierce, Thermo Fisher Scientific), and adjusted to equal protein concentrations. Laemmli’s buffer was added to the protein samples, followed by boiling at 95 °C for 5 min. Equal amounts of protein were separated by 7.5–15% gradient poly-acrylamide gel (REAL GEL PLATE, Bio Craft, Tokyo, Japan) electrophoresis and blotted onto polyvinyl difluoride membranes. The membranes were incubated with a blocking solution [Blocking One-P for Cx43 and Blocking One (Nacalai Tesque) for β-actin] and subsequently incubated with an anti-Cx43 Ab (1:10,000; rabbit polyclonal IgG; Abcam, Cambridge, UK) overnight at 4 °C or with an anti-β-actin Ab (1:20,000; clone AC-15, mouse monoclonal IgG1; Sigma-Aldrich) for 1 h at room temperature. After washing, the membranes were incubated with a horseradish peroxidase-conjugated secondary Ab for 1 h at room temperature. Then, the membranes were washed and visualized by enhanced chemiluminescence (ECL Prime, GE Healthcare Bio-Sciences AB, Uppsala, Sweden). Band intensities were measured using the ChemiDoc™ XRS system (Bio-Rad Laboratories, Hercules, CA, USA) and normalized to β-actin levels.

### Immunocytochemistry

Glial cells plated on collagen type 1-coated 8-well culture slides (Corning, Corning, NY, USA) were washed with PBS, fixed with 4% paraformaldehyde for 5 min, and permeabilised with 0.05% Triton X-100 in PBS (PBS-T) for 15 min. The cells were incubated with primary Abs against Cx43, Iba-1, GFAP, NeuN, Nogo-A, or NG2 (detailed information of Abs are listed in Supplementary Table S2) in PBS-T with 5% goat serum for 1 h at 37 °C. After rinsing, the cells were incubated with Alexa 488- and 546- or 594-conjugated secondary Abs and 4′,6-diamidino-2-phenylindole (DAPI) for 30 min at 37 °C. Images were captured using a confocal laser microscope system (Nikon A1, Nikon, Tokyo, Japan) with Plan-Apochromat 20 × (0.75 NA) or Plan-Apochromat 10 × (0.45 NA) objective (Nikon) or fluorescence microscope (BZ-X700, Keyence, Osaka, Japan) with Plan-Apochromat 20 × (0.75 NA) or Plan-Fluor 10 × (0.30 NA) objective (Nikon).

### RNA extraction and quantitative real-time reverse transcriptase (RT)-PCR

Total RNA was extracted from cells using an RNeasy Mini kit (Qiagen, Venlo, Netherlands) following the manufacturer’s instructions. cDNA was synthesized using ReverTra Ace qPCR RT Master Mix with gDNA Remover (Toyobo, Osaka, Japan). Quantitative real-time RT-PCR analysis of cDNAs was performed with an Applied Biosystems 7500 Real-Time PCR System (Applied Biosystems, Thermo Fisher Scientific) using TaqMan Gene Expression Master Mix and TaqMan Gene Expression Assays (Cx43 (*Gja1*), Mm01179639_s1; Gapdh, Mm99999915_g1; Applied Biosystems, Thermo Fisher Scientific). Gapdh was used as an internal control gene. PCR cycling conditions were 50 °C for 2 min, 95 °C for 10 min, followed by 40 cycles of 95 °C for 15 sec and 60 °C for 1 min. The ΔΔCT efficiency corrected method was used to calculate relative mRNA levels.

### SLDT Assay

GJ permeability was determined at 37 °C using an SLDT assay as described previously^59^ with minor modifications. In brief, confluent glial cells in 12-well plates were washed with PBS and scraped in the presence of PBS containing 0.1% of the fluorescent dye Lucifer yellow (molecular weight: 457.3 Da; Sigma-Aldrich) and 0.05% rhodamine B-dextran (molecular weight: 10,000 Da; Molecular Probes, Thermo Fisher Scientific). After 2 min of incubation, the cells were washed three times with PBS and then incubated for an additional 5 min in PBS to allow the loaded dye to transfer to adjoining cells. The cells were then fixed with 4% paraformaldehyde, counterstained with Hoechst 33342, and observed under a fluorescence microscope. Damaged cells absorb the dye mixture and transfer Lucifer yellow into neighbouring cells through functional GJs. In contrast, rhodamine B-dextran does not pass through GJ and is restricted to the initially loaded cells. Dye diffusion was captured using fluorescence microscope (BZ-X700, Keyence) with Plan-Fluor 10 × (0.30 NA) objective (Nikon), and quantified by measuring fluorescent areas. Quantification of the function of GJs was performed by subtraction of the rhodamine B-positive fluorescent area from the Lucifer yellow-positive fluorescent area by ImageJ software (US National Institutes of Health, Bethesda, MD, USA).

### ELISA

The levels of IFNγ in culture supernatants were measured by an ELISA kit (Quantikine^®^ ELISA Mouse IFN-γ Immunoassay; R&D systems) according to the manufacturer’s instructions.

### Multiplexed fluorescent bead-based immunoassay

IFNγ-treated microglia-conditioned media were collected and analysed simultaneously for 23 cytokines and chemokines: IL-1α, IL-1β, IL-2, IL-3, IL-4, IL-5, IL-6, IL-9, IL-10, IL-12 (p40), IL-12 (p70), IL-13, IL-17A, TNFα, granulocyte-colony stimulating factor, granulocyte-macrophage colony-stimulating factor, IFNγ, chemokine (C-X-C motif) ligand 1 (KC), chemokine (C-C motif) ligand (CCL) 2 (monocyte chemoattractant protein 1), CCL3 [Macrophage inflammatory protein (MIP)-1α], CCL4 (MIP-1β), CCL5 (RANTES), and CCL11 (eotaxin) by a Bio-Plex Multiplex System (Bio-Rad Laboratories) according to the manufacturer’s instructions^36^. All samples were analysed undiluted in duplicate.

### Statistical analysis

Data are expressed as the mean ± standard deviation (s.d.) of at least four experiments. One-way analysis of variance (ANOVA) followed by a Dunnett’s multiple comparison test were used to analyse data. All analyses were carried out using JMP^®^ Pro version 11.0.0 software (SAS Institute, Cary, NC, USA). The significance level was set at *p* < 0.05.

## Additional Information

**How to cite this article**: Watanabe, M. *et al*. Th1 cells downregulate connexin 43 gap junctions in astrocytes via microglial activation. *Sci. Rep.* **6**, 38387; doi: 10.1038/srep38387 (2016).

**Publisher's note:** Springer Nature remains neutral with regard to jurisdictional claims in published maps and institutional affiliations.

## Supplementary Material

Supplementary Information
