# Supplementary material for: Th1 cells downregulate connexin 43 gap junctions in astrocytes via microglial activation
Source: Sci Rep. 2016 Dec 8;6:38387. doi: 10.1038/srep38387 (PMC5143974; doi:10.1038/srep38387)
Supplement: Supplementary Information [file srep38387-s1.pdf]

Th1 cells downregulate connexin 43 gap junctions in astrocytes via microglial activation

Mitsuru Watanabe, MD,[1] Katsuhisa Masaki, MD, PhD,[1] Ryo Yamasaki, MD, PhD,[1] Jun Kawanokuchi, PhD,[2,3] Hideyuki Takeuchi, MD, PhD,[2,4] Takuya Matsushita, MD, PhD,[1] Akio Suzumura, MD, PhD,[2] Jun-ichi Kira, MD, PhD[1,*]

[1] Department of Neurology, Neurological Institute, Graduate School of Medical Sciences, Kyushu University, Fukuoka 812-8582, Japan

[2] Department of Neuroimmunology, Research Institute of Environmental Medicine, Nagoya University, Nagoya 464-8601, Japan

[3] Institute of Traditional Chinese Medicine, Suzuka University of Medical Science, Suzuka 510-0226, Japan

[4] Department of Neurology and Stroke Medicine, Yokohama City University Graduate School of Medicine, Yokohama 236-0004, Japan

Supplementary Figures

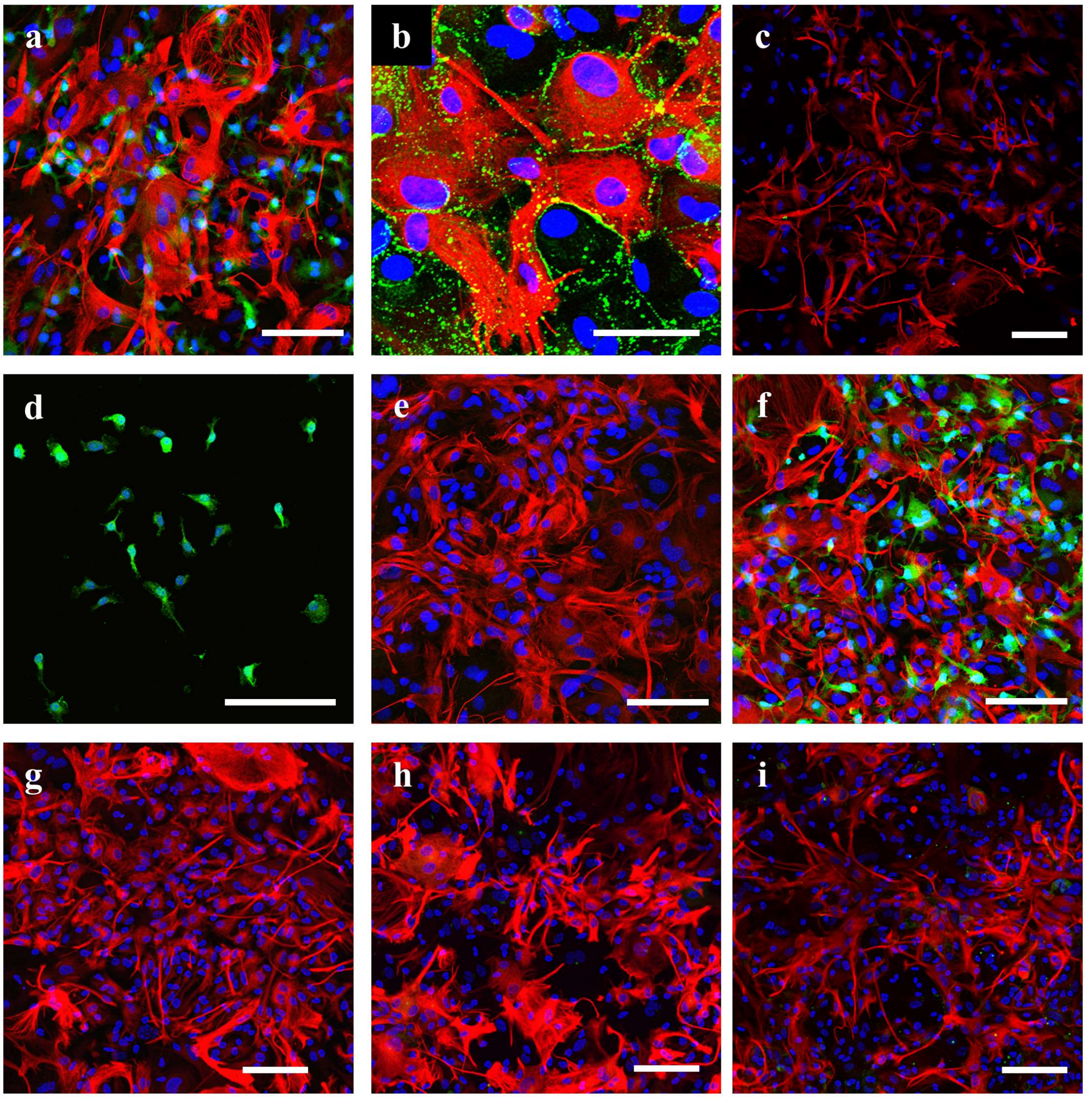

Supplementary Figure S1. Identification of cell types and expression of Cxs in mixed and purified glial cell cultures. (a, d–f) Cells in primary mixed glial cell cultures (a), microglial cell cultures (d), astrocyte-rich cultures (e), and astrocyte/microglia-mixed cultures (f) were fixed and immunostained for Iba-1 (green) and GFAP (red), and counterstained with DAPI (blue). (b, c) Cells in primary mixed glial cell cultures were fixed and immunostained for Cx43 (green) (b) or Cx30 (c) and GFAP (red), and counterstained with DAPI (blue). (g–i) Cells in primary mixed glial cell cultures were fixed and immunostained for NeuN (green) (g), Nogo-A

(green) (h), or NG2 (green) (i) and GFAP (red), and counterstained with DAPI (blue).

Scale bars: a, c–i, 100 μm; b, 50 μm.

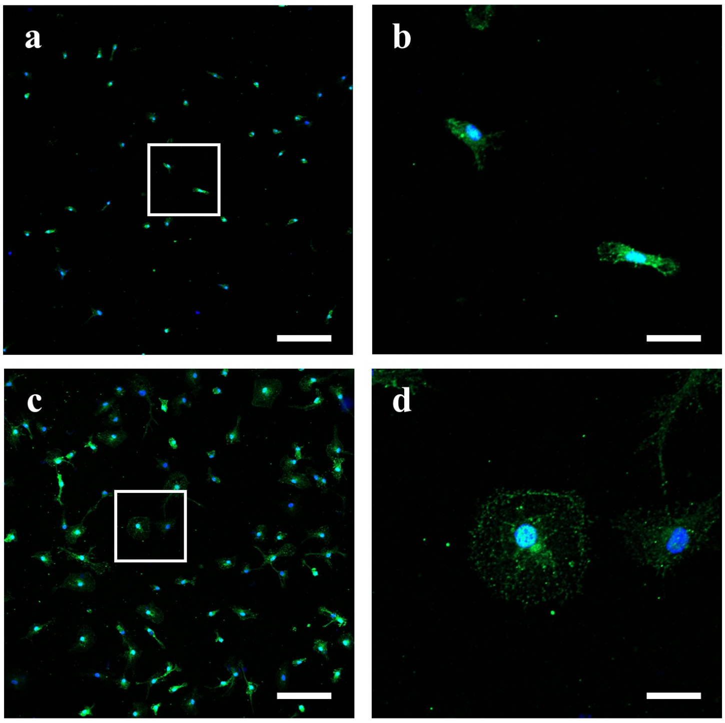

Supplementary Figure S2. Morphological changes of microglia induced by IFNγ treatment. Microglial cell cultures treated with the vehicle (a, b) or 500 ng/ml IFNγ (c, d) for 24 h were fixed and immunostained for Iba-1 (green) and counterstained with DAPI (blue). (a, b) Microglia treated with the vehicle (resting microglia) showed a rod shape. Panel b shows a higher magnification image of the boxed area in (a). (c, d) Microglia treated with 500 ng/ml IFNγ showed a large round amoeboid shape. Panel d shows a higher magnification image of the boxed area in (c). Scale bars: a and c, 100 μm; b and d, 20 μm.

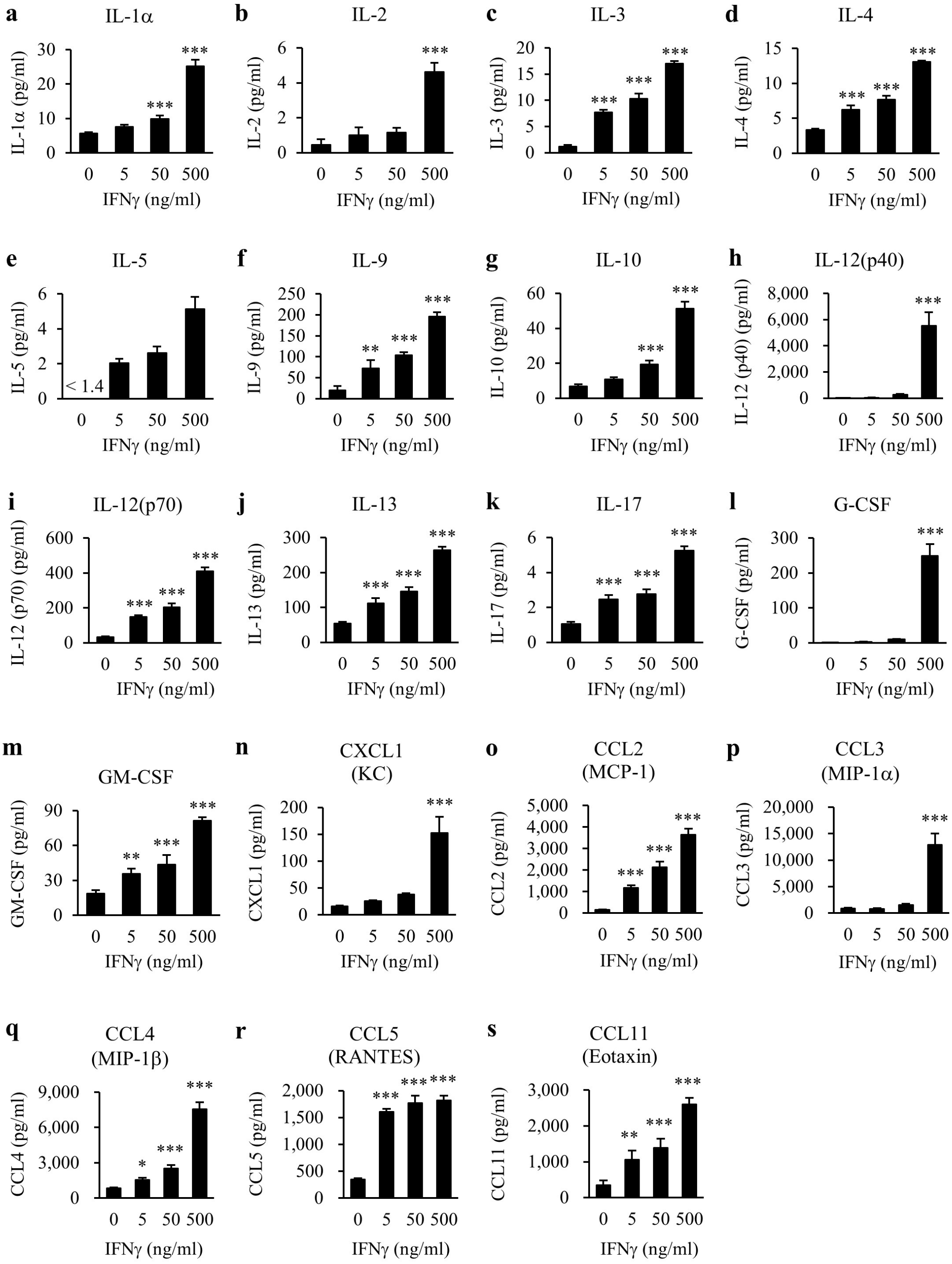

Supplementary Figure S3. Cytokine and chemokine concentrations in IFNγ-treated microglia-conditioned media. Isolated microglia were treated with the vehicle (0) or 5, 50, and 500 ng/ml IFNγ for 24 h. IFNγ-treated microglia-conditioned media were collected, and cytokine and chemokine concentrations were measured by

the Bio-Plex Multiplex System. The expression level of each cytokine and chemokine is shown in each graph. Data are presented as the mean ± s.d. (n = 4, * $p < 0.05$, ** $p < 0.01$, and *** $p < 0.001$, compared with the vehicle-treated control by one-way ANOVA followed by Dunnett's multiple comparison test).

Abbreviations (not stated in the main text): CXCL, chemokine (C-X-C motif) ligand; G-CSF, granulocyte-colony stimulating factor; GM-CSF, granulocyte-macrophage colony-stimulating factor.

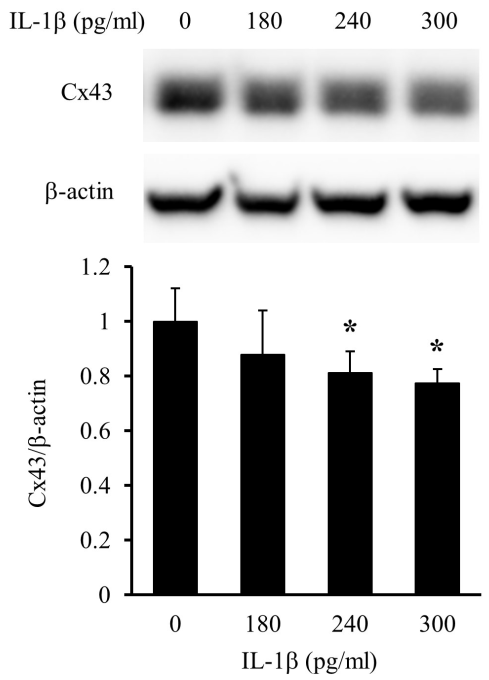

Supplementary Figure S4. Dose-dependent reduction of Cx43 protein levels by IL-1β treatment in astrocyte-rich cultures. Astrocyte-rich cultures were treated with the vehicle (0) or 180, 240, and 300 pg/ml IL-1β for 24 h. Cx43 protein levels were evaluated by western blotting. β-actin was used as a loading control. Cx43/β-actin of the vehicle treatment was set as 1. Data are presented as the mean ± s.d. (n = 5, * $p < 0.05$, compared with the control by one-way ANOVA followed by Dunnett's multiple comparison test). Full-length blots are presented in Supplementary Figure S10.

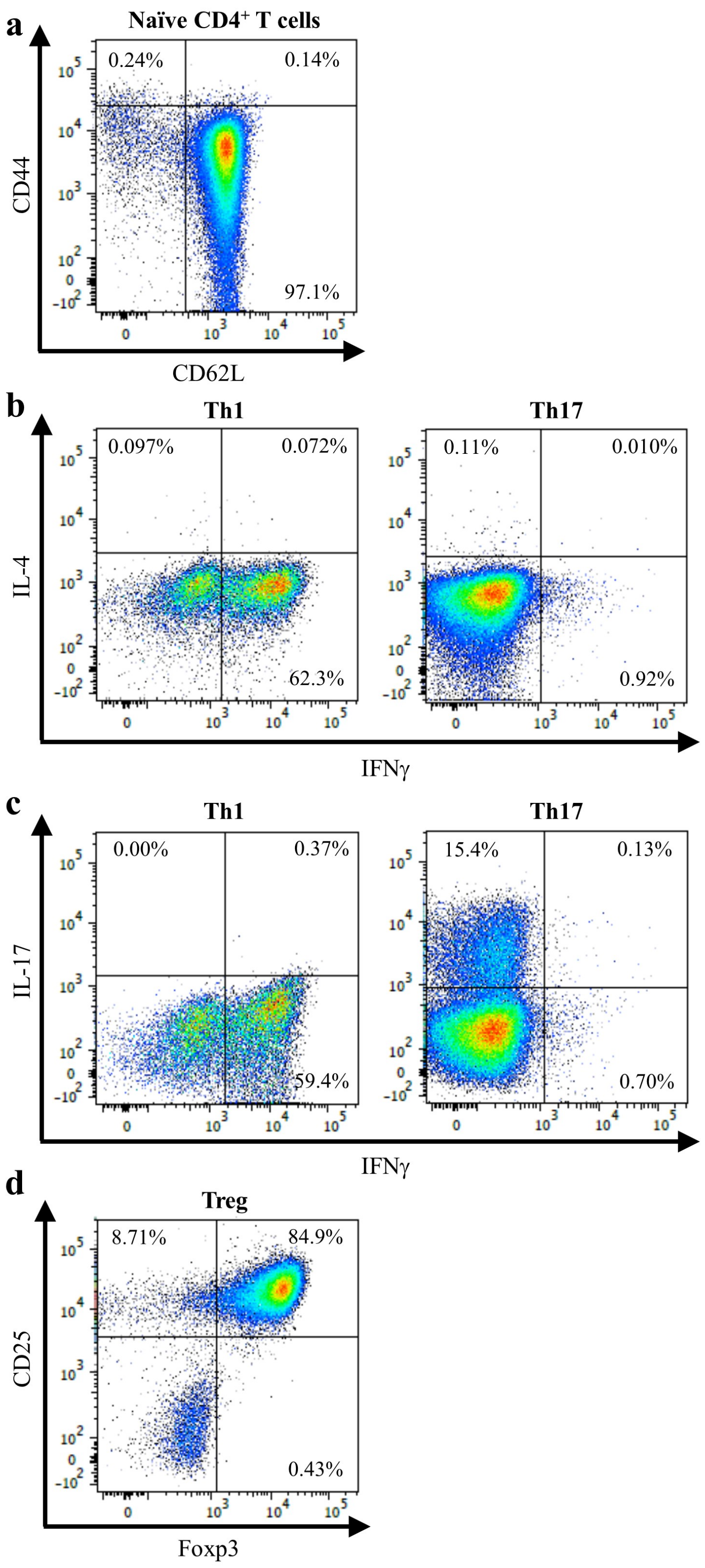

Supplementary Figure S5. Flow cytometric analysis of differentiated CD4$^+$ T cell subsets *in vitro*. (a) Naïve CD4$^+$ T cells were isolated from mouse splenocytes using a

Naïve $CD4^+$ T Cell Isolation Kit. The purity of naïve $CD4^+$ T cells ($CD4^+CD44^{lo}CD62L^+$) was determined by flow cytometry. (b, c) Flow cytometric analyses of IFNγ and IL-4 expression (b), and IFNγ and IL-17 expression (c) in Th1 and Th17 cells differentiated from naïve $CD4^+$ T cells *in vitro*. These Th1 and Th17 cells were well differentiated and produced only subset-specific cytokines. (d) Assessment of Treg cells ($CD4^+CD25^+Foxp3^+$) differentiated from naïve $CD4^+$ T cells *in vitro* by flow cytometric analysis. Representative data are shown.

Fig.1(a)

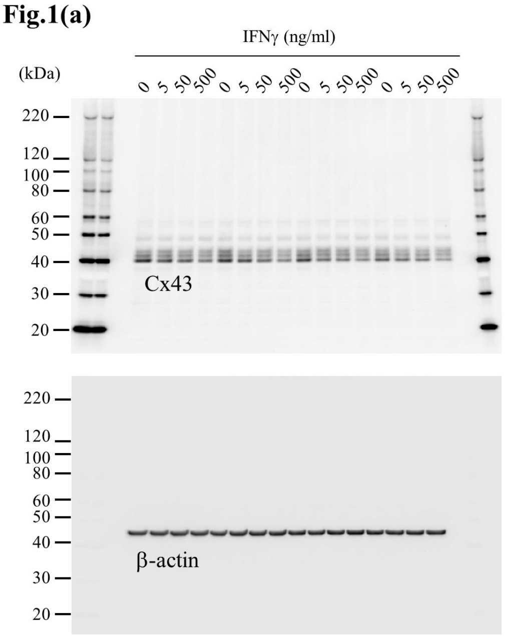

Fig.1(b)

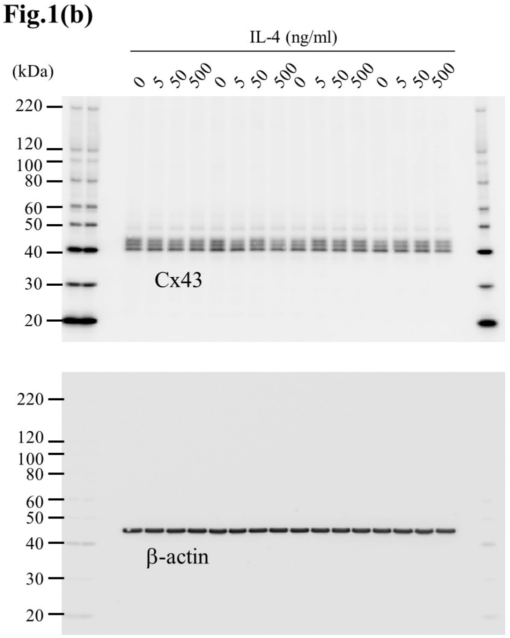

Fig.1(c)

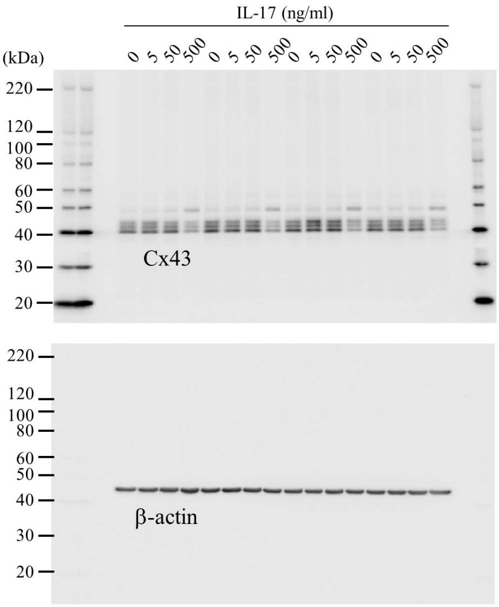

Supplementary Figure S6. Full-length immunoblots of Figure 1.

Fig.2(a)

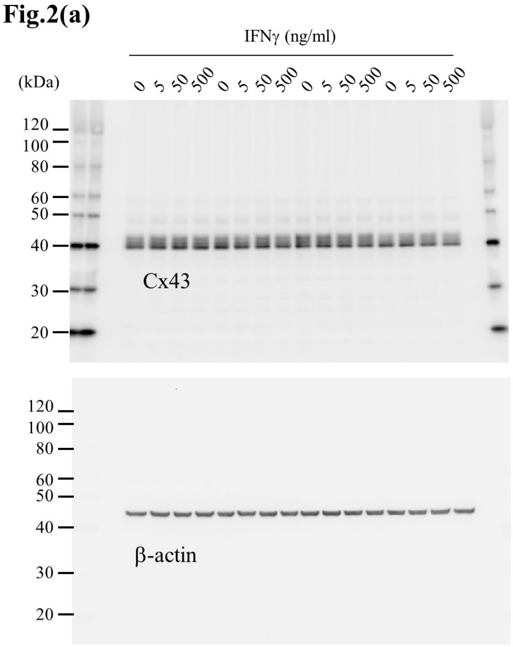

Fig.2(b)

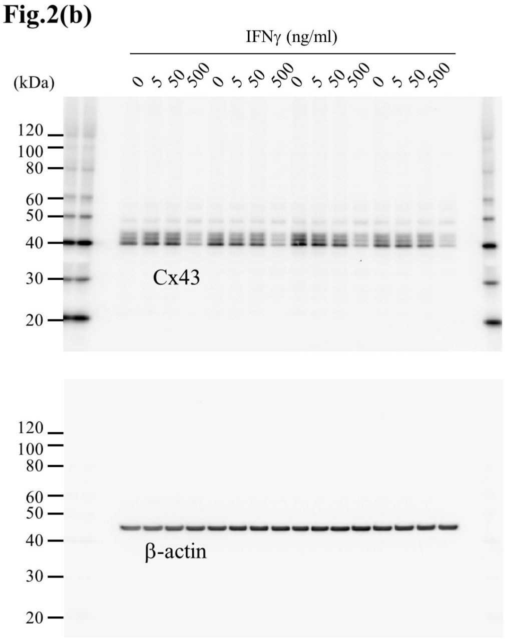

Fig.3(a)

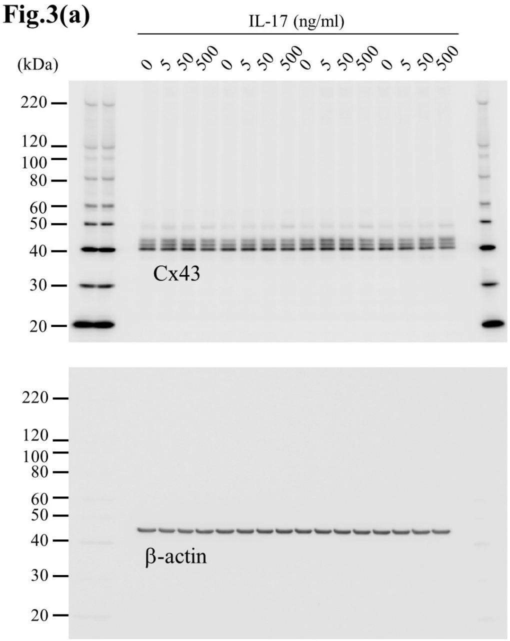

Fig.3(b)

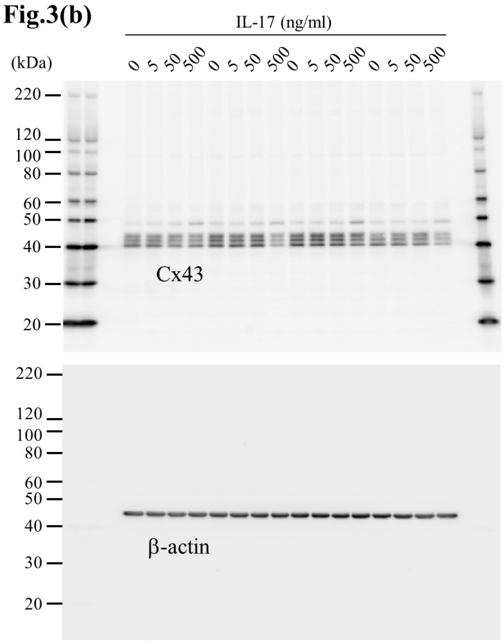

Supplementary Figure S7. Full-length immunoblots of Figures 2 and 3.

Fig.5

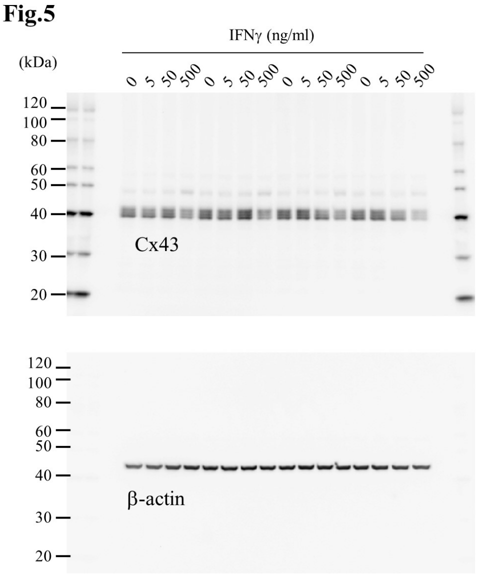

Fig.7(a)

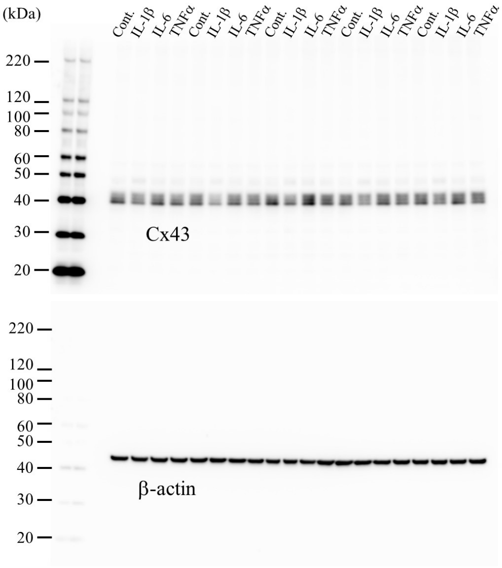

Fig.7(b)

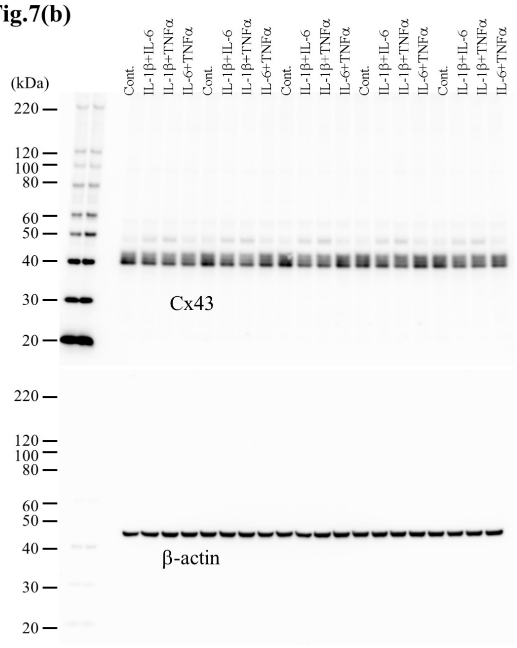

Supplementary Figure S8. Full-length immunoblots of Figures 5 and 7.

Fig.8

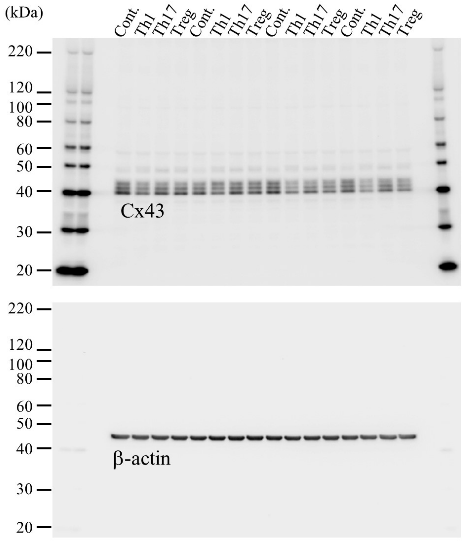

Supplementary Figure S9. Full-length immunoblots of Figure 8.

Supplementary Fig. S4

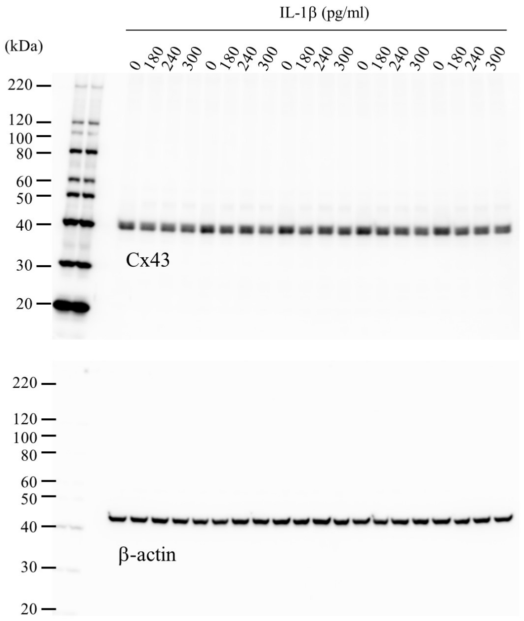

Supplementary Figure S10.

Full-length immunoblots of Supplementary Figure S4.

Supplementary Table S1. Antibodies used for flow cytometry.

Antigen	Clone	Type	Source
CD4 (mouse)	RM4-5	Rat IgG2a, κ	Sony Biotechnology (Champaign, IL, USA)
CD4 (mouse)	GK1.5	Rat IgG2b, κ	eBiosciences (San Diego, CA, USA)
CD16/32 (mouse)	93	Rat IgG2a, λ	Sony Biotechnology (Champaign, IL, USA)
CD25 (mouse)	PC61	Rat IgG1, λ	Sony Biotechnology (Champaign, IL, USA)
CD44 (mouse/human)	IM7	Rat IgG2b, κ	Sony Biotechnology (Champaign, IL, USA)
CD62L (mouse)	MEL-14	Rat IgG2a, κ	Sony Biotechnology (Champaign, IL, USA)
I-A/I-E (mouse)	M5/114.15.2	Rat IgG2b, κ	BioLegend (San Diego, CA, USA)
IFNγ (mouse)	XMG1.2	Rat IgG1, κ	Sony Biotechnology (Champaign, IL, USA)
IL-4 (mouse)	11B11	Rat IgG1, κ	Sony Biotechnology (Champaign, IL, USA)
IL-17A (mouse)	TC11-18H10.1	Rat IgG1, κ	Sony Biotechnology (Champaign, IL, USA)
Foxp3 (mouse/rat)	FJK-16s	Rat IgG2a, κ	eBiosciences (San Diego, CA, USA)

Supplementary Table S2. Antibodies used for immunocytochemistry.

Antigen	Type	Dilution	Source
Cx43	Rabbit polyclonal IgG	1:1,000	Abcam (Cambridge, UK)
GFAP	Mouse monoclonal IgG1 (clone GA5)	1:1,000	Merck Millipore (Darmstadt, Germany)
GFAP	Rabbit polyclonal IgG	1:1,000	Dako, Agilent Technologies (Glostrup, Denmark)
Iba-1	Rabbit polyclonal IgG	1:1,000	Wako (Osaka, Japan)
NeuN	Mouse monoclonal IgG1 (clone A60)	1:1,000	Merck Millipore (Darmstadt, Germany)
Nogo-A	Rabbit polyclonal	1:1,000	Abcam (Cambridge, UK)
NG2	Rabbit polyclonal	1:200	Merck Millipore (Darmstadt, Germany)
